# Quantitative functionalization of biosynthetic caged protein materials

**DOI:** 10.15302/J-QB-022-0306

**Published:** 2023-03-01

**Authors:** Quan Cheng, Xuan Wang, Xian‐En Zhang, Chengchen Xu, Feng Li

**Affiliations:** ^1^ College of Life Sciences and Health Wuhan University of Science and Technology Wuhan 430065 China; ^2^ State Key Laboratory of Virology Wuhan Institute of Virology Center for Biosafety Mega‐Science Chinese Academy of Sciences Wuhan 430071 China; ^3^ Faculty of Synthetic Biology Shenzhen Institute of Advanced Technology Chinese Academy of Sciences Shenzhen 518055 China; ^4^ National Laboratory of Biomacromolecules Institute of Biophysics Chinese Academy of Sciences Beijing 100101 China; ^5^ University of Chinese Academy of Sciences Beijing 100049 China

**Keywords:** protein nanocages, virus‐like particles, functionalization, genetic modification, chemical modification

## Abstract

**Background:**

As one of the representative protein materials, protein nanocages (PNCs) are self‐assembled supramolecular structures with multiple advantages, such as good monodispersity, biocompatibility, structural addressability, and facile production. Precise quantitative functionalization is essential to the construction of PNCs with designed purposes.

**Results:**

With three modifiable interfaces, the interior surface, outer surface, and interfaces between building blocks, PNCs can serve as an ideal platform for precise multi‐functionalization studies and applications. This review summarizes the currently available methods for precise quantitative functionalization of PNCs and highlights the significance of precise quantitative control in fabricating PNC‐based materials or devices. These methods can be categorized into three groups, genetic, chemical, and combined modification.

**Conclusion:**

This review would be constructive for those who work with biosynthetic PNCs in diverse fields.

## Abstract 2

**Author summary:** Protein nanocages (PNCs) are a group of self‐assembling biomacromolecules that have been recognized as natural nanomaterials and are attracting increasing interest in multidisciplinary fields. Various strategies have been developed to modify and functionalize PNCs for tailored applications. This review aims to summarize the currently available methods for PNC modification with emphasis on the significance of quantitative control of functionalization.

## INTRODUCTION

**Figure 1:**
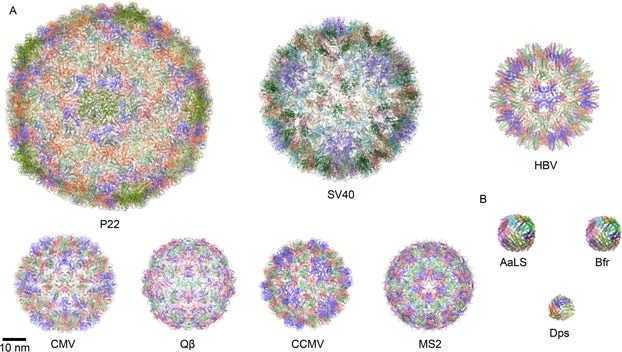
**Examples of PNCs.** (A) The structural models of representative VLPs. Bacteriophage P22 (P22, PDB ID: 5UU5), simian virus 40 (SV40, PDB ID: 1SVA), hepatitis B virus (HBV, PDB ID: 6UI7), cucumber mosaic virus (CMV, PDB ID: 5OW6), bacteriophage Qbeta (Qβ, PDB ID: 5VLY), cowpea chlorotic mottle virus (CCMV, PDB ID: 1ZA7), and bacteriophage MS2 (MS2, PDB ID: 6RRS). (B) The structural models of representative non‐viral PNCs. *Aquifex aeolicus* lumazine synthase (AaLS, PDB ID: 1HQK), bacterioferritin (Bfr, PDB ID: 1BFR), and DNA‐binding protein from starved cells (Dps, PDB ID: 1QGH).

Protein nanocages (PNCs) are self‐assembled supramolecular structures composed of highly ordered protein subunits [1]. With typical diameters in the range of 10‒200 nm, PNCs can be divided into two categories by the structural component ( Fig.1): virus‐like particles (VLPs) formed by viral capsid proteins [2, 3, 4, 5] and non‐viral PNCs [6,7]. PNCs usually have an inner cavity, an outer surface, and interfaces between subunits. The interior presents the load‐carrying capacity, the interactions between subunits control the disassembly and assembly, and the outer surface is often related to the immunogenicity and molecular recognition ability [8]. As natural nanomaterials, PNCs can be synthesized and engineered in various expression systems including *Escherichia coli (E. coli)*, yeast, plant, insect cells, mammalian cells and cell‐free *in vitro* translation systems. Thanks to the advantageous features such as good monodispersity, biocompatibility, structural addressability, and facile production, PNCs have been widely explored in catalysis [9], biosensing [10], bioimaging [11], drug delivery [12], and disease diagnosis and treatment [13].

## SIGNIFICANCES OF PRECISE QUANTITATIVE FUNCTIONALIZATION OF PNCS

As a group of proteins with highly ordered architectures, PNCs have a variety of modifiable groups, which together with the inner cavity serve as the sites or space for modification and functionalization. The modification degree of PNCs is a critical index impacting the gained function and the stability of PNCs. Precise quantitative functionalization is considered an optimal and predictable way to achieve expected functions. A precise quantitative procedure, on the other hand, is also necessary for a valuable record that is experimentally repeatable.

Here are two examples highlighting the significance of precise quantitative functionalization. Glycosylation is one of the typical functionalization methods in VLPs design [14,15]. In the glycosylation of VLPs composed of the smallest surface antigen (HBsAg) of HBV, the mutation introducing one additional glycosylation site (T116N) increased glycosylation and immunogenicity in comparison with the wild type VLPs [16,17]. Similarly, antibodies were elicited by linking M2e (the outer domain of influenza virus A M2 protein) to the N terminus of the VLPs assembled from the HBV core antigen (HBcAg). The copy number of M2e was negatively correlated with the produced HBcAg antibody level but positively correlated with the M2e‐specific antibody IgG1 level produced after the first injection [18]. In the construction of a lentiviral system for mRNA delivery, a stem‐loop signal sequence mediated the mRNA packaging. The larger the copy number of the signal, the more efficient the mRNA‐specific packaging. But too large copy number could make the coding plasmid unstable and occupy the cargo space. A balance (6 copies) was finally achieved to guarantee efficient packaging while avoiding the plasmid stability problem [19].

**Figure 2:**
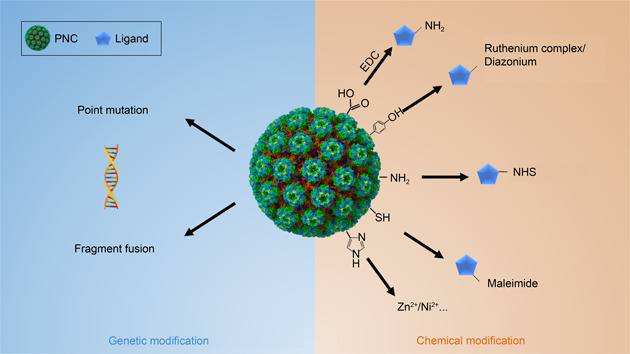
Methods for functionalizing PNCs.

The examples above demonstrate the significant effects of quantitative modification on the functions of VLPs. In these studies, quantitative analysis of the functionalized motifs and the corresponding outcomes are essential to correlating quantitative functionalization with biological effects so that an optimized extent of modification can be figured out to meet a designed function. Therefore, quantitative modification of PNCs and relevant analysis techniques are worthy of considerable investigation. In this review, we summarize the functionalization methods of PNCs from a quantitative point of view and highlight the significance of precise quantitative control of functionalization in varied application scenes ( Fig.2).

## STRATEGIES FOR QUANTITATIVE FUNCTIONALIZATION OF PNCS

### Genetic modification

Genetic engineering is a major approach to precisely introduce changes into PNCs [2,20,21]. Generally, genetic modification can change the coding sequence of PNC subunits through point mutation, deletion, or insertion to modify the primary amino acid sequence, resulting in the integration of functional modules in the higher‐organized PNC structures. Based on the central dogma, genetic engineering is a strictly precise way of quantitative functionalization of PNCs. Several genetic modification strategies that are commonly utilized in PNCs are summarized in the following.

#### Point mutation

Point mutation is often performed for residue substitution. In a recent study, mutation of lysine 13 to glutamate (K13E) in bacteriophage Qβ VLPs, which consists of 180 identical subunits, resulted in a marked increase in cellular binding. In contrast, mutation of lysine 46 to glutamine (K46Q) resulted in a significant reduction in mammalian cell binding [22]. In this work, quantitative modification of PNCs by point mutation can reduce the non‐specific binding with cells, and non‐binding variants after modification are promising materials for targeting studies. As a fundamental approach of genetic engineering, point mutation can not only regulate cellular recognition but also install possible motifs for further functionalization through chemical modification, which is discussed in the section of “combination of genetic and chemical modification”.

**Figure 3:**
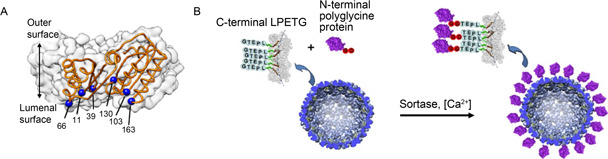
**Genetic modification of PNCs.** (A) Arginine mutations (blue spheres) presented on a monomer of OP mutant PNCs [23]. Copyright 2018 American Chemical Society. (B) The attachment between the N‐terminal glycine of the protein (purple) and C‐terminal threonine of the P22 VLPs (blue) with sortase [24]. Copyright 2017 American Chemical Society.

The charge state of PNC’s inner cavity depends on the charged residues on the inner surface. Point mutation can be utilized to alter the charge state of the inner surface to tune the loading efficiency of PNCs. Edwardson *et al*. took O3‐33, a non‐viral PNC, as a starting template for functional modification. A variant OP was decorated with a large number of positive charges in the interior by replacing the residues Thr11, Pro39, Glu66, Trp103, Phe130, and Leu163 of O3‐33 with arginine ( Fig.3). The resultant OP can encapsulate single‐stranded (ss) RNA, double‐stranded (ds) DNA, and dsRNA, with each OP encapsulating two oligonucleotides. Furthermore, to examine the delivery efficiency of OP, small interfering RNA (siRNA) was encapsulated as the model cargo toward HeLa cells, and induction of RNA interference (RNAi) and downregulated gene expression were achieved [23]. Precise modification of charge state has been proven to be an effective way in the design of PNCs for encapsulation of nucleic acids or other cargoes that have electrostatic interactions with the inner surface.

#### Fragment fusion

Fragment fusion involves the insertion of a peptide into a specific region or linkage of a peptide to the N‐ or C‐terminus of the PNC subunits. In such functional engineering, particle stability and modification yield of PNCs are often hard to predict. The N‐terminal region of bacteriophage MS2 capsid protein is confined and can only be modified limitedly, hindering the possibility of N‐terminal extension [25]. Brauer *et al*. constructed a P‐X‐X‐X‐MS2 library by replacing the methionine residue at the N terminus of wild‐type MS2 capsid protein with a short fragment P‐X‐X‐X (X being any amino acid residue). Three percent out of the 8,000 library variants presented expected performance after screening for self‐assembly, thermal stabilization, and functional modification. During functional selection, modification efficiencies on both interior surface with the AlexaFluor‐488 dye [26—28] and exterior surface with tyrosinase from *Agaricus bisporus* were close to 100% [29]. This study describes nonintuitive design rules governing N‐terminal short fragment fusion with high modification potential.

In vaccine design and development, exogenous epitope display on PNCs *via* fusing short fragments is a common method to change the immunogenicity. However, if the fusion site or the properties of the fragment are not suitable, self‐assembly of PNCs could probably be “disabled”. Computer‐aided analysis and prediction can deal with such problems to some extent. In a study as early as 2000, the e1 loop of HBcAg was set to be the insertion site, and the effects of inserting fragments with different chemical and physical properties on the assembly of PNCs were predicted. As a result, when inserted epitopes have a large size, high hydrophobicity, or high β‐strand index, the self‐assembly capacity of the VLPs would be influenced [30].

Fragment fusion on the surface of PNCs can contribute to the binding specificity toward host cells. For instance, Takahashi *et al*. designed a flag tag flanked by glycine spacers (GGG—DYKDDDDK—GGG) to replace the amino acid residues 137—138 at the DE loop exposed on the surface of SV40 VLPs so that the VLPs could reduce natural virus‐cell interaction while retaining the packaging ability. Only a limited number of residues, such as residues 137—138 on the DE loop and residues 272—275 on the HI loop, were selected as appropriate candidates for fragment fusion while maintaining the self‐assembly properties of SV40 VLPs [31].

In order to break through the restriction of the size and structure of heterogeneous antigens in the formation of PNCs, genetic modification can also fuse different epitopes at the same site on PNCs. For example, a combination of polyepitope VP1 (residues 141—160) and VP4 (residues 21—40) from foot‐and‐mouth disease virus (FMDV) was inserted into the 75—82 residues of HBcAg VLPs. Peptide‐specific antibodies were characterized after mouse immunization by indirect ELISA (iELISA). The chimeric VLPs with insertion of two epitopes showed higher immunogenicity and elicited higher‐titer antibodies with high specificity compared to the VLPs inserted with only one epitope [32].

For installing an intact domain or protein onto PNCs, direct fusion often leads to failure in protein folding or PNC assembly due to steric hindrance. An alternative way is the fusion of short peptides, which serve as anchors for linking functional domains or proteins. For example, P22 VLPs have been developed as catalytic and therapeutic nanomaterials [33]. Dustin *et al*. introduced a modular method for covalently linking protein domains to the exterior of P22 VLPs through an enzyme‐mediated linkage ( Fig.3). The *Staphylococcus aureus* sortase was used for covalently pairing the recognition sequence of the C‐terminal LPETG peptide with the N‐terminal polyglycine sequence [34]. Anchor‐dependent enzymatic linking of proteins can significantly increase the efficiency of protein cargo loading in PNCs. In a study by Schoonen *et al.*, CCMV‐ELP (elastin‐like polypeptide) was linked with a green fluorescent protein (GFP) tagged by LPETG peptide using sortase, resulting in a GFP encapsulation efficiency of 16—18 peptides per capsid, which was higher than the non‐covalent encapsulation [24,35,36]. Another popular genetic tool for attaching intact domain or protein onto PNCs is the SpyTag/SpyCatcher system. Concerning this system, comprehensive reviews can be referred [37,38].

Taken together, genetic modification is a powerful way for introducing new functions into PNCs with precisely quantitative control. Genetic modification of PNCs is easy to carry out in virtue with genetic cloning techniques and strategies from synthetic biology. This is an advantage of the biosynthetic materials, PNCs, over chemically synthesized macromolecules. However, genetic modification sometimes leads to failure in expression, correct folding, and solubility of PNCs. Further endeavors and technological breakthrough are needed to enable fully predictable genetic modification of PNCs.

### Chemical functionalization

Chemical modification is also an essential group of approaches for PNC functionalization. It involves linking functional molecules or motifs to specific sites of PNCs through chemical reactions. A group of chemical methods summarized below has been applied to introduce functional modules to the interior, exterior, or N/C terminus of PNCs, such as phosphorylation, acetylation, glycosylation, amidation, *etc*. These chemical reactions exhibit significant control with possibilities of different functionalization, which contributes to the diversity of PNCs.

#### Covalent functionalization with cysteine

Cysteine contains a sulfhydryl group and can form covalent bonds efficiently with other sulfhydryl‐containing residues or maleimide, which offers a handle for functionalization. Cysteine is usually not abundant in proteins. Some proteins even have no cysteine. Thus, cysteine is an ideal residue for quantitative and site‐specific modification of PNCs. Ambuhl *et al*. developed a vaccine for hypertension with Qβ VLPs modified with angiotensin II‐derived peptides. Specifically, an average of two to three angiotensin II peptides were conjugated to each Qβ VLP subunit by covalent linking between cysteines from the peptides and the lysines on the Qβ VLPs using a bivalent cross‐linker. In this study, the cysteine residue brings the angiotensin II‐derived peptide onto the Qβ VLP surface, resulting in a vaccine that reduced the blood pressure in the immunized hypertensive rats [39,40]. Another example is linking anti‐PD‐1 peptides onto the surface of the VLPs of CPMV, a non‐enveloped plant virus that is non‐infectious but immunogenic to mammals. Anti‐PD‐1‐decorted CPMV VLPs were constructed *via* connecting the anti‐PD‐1 peptides to the NHS arms on CPMV VLPs in virtue with the C‐terminal cysteines of the anti‐PD‐1 peptide. Quantitative densitometry revealed that 24—27 anti‐PD‐1 peptides were linked onto each CPMV VLP. The anti‐PD‐1‐decorted CPMV VLPs presented increased antitumor efficacy in the tumor mouse model [41]. If there is no exposed cysteine on the outer surface of PNCs, it will be convenient to site‐specifically introduce cysteines with controlled numbers on demand. For example, Anand *et al.* developed a drug delivery system based on P22 VLPs. The VLPs were decorated with a fluorescence‐labeled cell‐penetrating peptide on the outer surface through an introduced cysteine residue and were loaded with the analgesic agent Ziconotide (Prialt^®^). Densitometric analysis revealed that about 40% of the capsid protein was labeled by the cell‐penetrating peptide, while about 543 Ziconotide peptides were encapsulated in each VLP [42]. This multi‐functional P22 VLP plays a potential role in brain drug delivery across the blood‐brain barrier.

#### Coupling with primary amine groups of lysine

Lysine is an amino acid carrying a primary amino group in the side chain that can be coupled explicitly with chemical conjugation reagents such as N‐hydroxysuccinimide (NHS), isothiocyanate [43, 44, 45, 46]. Ashley *et al*. simultaneously displayed SP94 (SFSIIHTPILPL)‐specific targeting peptide [47,48] and histidine‐rich H5WYG peptide on the ricin‐loaded MS2 VLPs. Each functional peptide was decorated with a cysteine residue at the C terminus and connected to the lysine residue on the MS2 surface *via* a PEG linker that targeted both the sulfhydryl group of cysteine and the amino group of lysine. It was verified by quantitative analysis that the most effectively functionalized MS2 VLPs were decorated with about 60 SP94 peptides and 75 H5WYG peptides, which can almost eliminate the human hepatocellular carcinoma (HCC) cell line, Hep3B, without interacting with non‐targeted cells (such as hepatocytes) [49].

Hydrazones are a class of organic compounds formed by the condensation of carbonyl and hydrazine. Brunel *et al*. utilized hydrazone linkage to the assembly of CPMV VLPs. Firstly, lysine residues on the surface of CPMV VLPs were covalently modified with benzaldehyde. Then the hydrazide group of F56f (a high‐affinity peptide that can inhibit the growth and metastasis of cancer cells) ligand reacted specifically with some benzaldehydes on the VLPs. Finally, a PEG‐peptide ended with a hydrazide group was attached to the remaining benzaldehyde sites on the VLPs, resulting in the bi‐functionalized CPMV VLPs. The decorated F56f peptide effectively targeted the CPMV VLPs to endothelial cells, and the PEG‐peptides significantly reduced non‐specific binding. By quantitative reactions of 2‐hydrazinopyridine, the number of lysine residues that were modified with benzaldehydes was 280 out of 300 per VLP [46].

**Figure 4:**
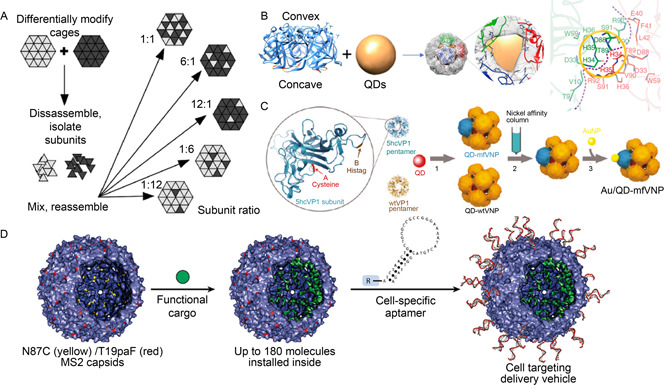
**Quantitative functionalization of PNCs through chemical modification.** (A) Assembly of multifunctionalized particles in different ratios of subunits [45]. Copyright 2020 Wiley‐VCH. (B) His‐tag was inserted between D33 (red) and G34 (yellow) of CcmL subunit for metal coordination [50]. Copyright 2022 PNAS. (C) Monofunctionalization of PNCs using affinity chromatography in virtue of introduced polyhistidine [51]. Copyright 2011 American Chemical Society. (D) The synthetic strategy of dual‐surface‐modified VLPs for targeted delivery [27]. Copyright 2009 American Chemical Society.

After lysine‐mediated modification, PNCs can be further quantitatively functionalized through reversible self‐assembly. For example, two groups of individually modified CCMV VLPs by biotin or digoxigenin ligands that respectively decorated on lysine residues were firstly disassembled to generate two kinds of subunits, biotin‐labeled and digoxigenin‐labeled dimers. Then the biotin‐ or digoxigenin‐labeled dimers were mixed at various ratios from 1:12 to 12:1 for reassembly to create differently functionalized CCMV VLPs ( Fig.4) [45]. The disassembly/reassembly strategy provides a rational approach to precisely regulating the numbers of different functional ligands on PNCs to reach the optimal ratio.

Through modification, not only PNCs’ properties can be changed, but also PNCs can optimize the properties of the ligands. In biological applications, a major disadvantage of C_60_ is insolubility in water. Linking derivatives of C_60_ to PNCs generated water‐soluble complexes. In particular, the fullerene derivative [6,6]‐phenyl‐C_61_‐butyric acid (PCBA) was activated by carbodiimide‐N‐hydroxysuccinimide chemistry and linked to CPMV VLPs *via* the solvent‐exposed Lys38 residues [52]. This complex holds potential in biomedicine and exemplifies a route to the water‐solubilization of hydrophobic drugs.

#### Carboxyl‐based functionalization of aspartic acid/glutamate

The carboxylate groups of aspartic acid and glutamates are usually applied for covalent functionalization on PNCs. However, because of high abundance of aspartic acid/glutamate in proteins, it is generally difficult to achieve precise quantitative functionalization *via* chemical modification on carboxylate groups of PNCs. Doxorubicin (DOX), one of the most widely studied chemotherapeutic agents, is a model drug for evaluating the delivery efficiency of PNCs. To enhance the cytotoxicity of DOX, Evans group cross‐linked eighty DOX molecules onto aspartic acids and glutamates on the surface of each CPMV VLP using 1‐ethyl‐3‐(3‐dimethylaminopropyl) carbodiimide hydrochloride (EDC) and NHS [53]. Installing redox centers onto the CPMV VLPs could generate a potential material for biosensing and catalytic applications. Taking the advantage of abundant carboxylate groups on CPMV VLP surface, Evans group loaded the ferrocene with a short length onto the VLPs. The number of ferrocenes on each VLP was determined to be approximately 174 by electrochemical methods. The ferrocenes modified on the exterior of the CPMV VLPs act as a multi‐electron reservoir, while the VLPs provide the redox‐active components [54].

#### Metal complexation with imidazole group of histidine

The imidazole group of histidine has the unique property of chelating with divalent metal ions. Recently, our group and collaborators have designed a novel PNC assembled from the β‐carboxysome shell vertex protein, CcmL, using the CdSe/ZnS quantum dots (QDs) as a template. A polyhistidine tag (His‐tag) was inserted between the residues D33 and G34 to introduce the interactions between imidazole rings and metal ions ( Fig.4) to promote the assembly. As was analyzed by the biofilm layer interference technology (BLI), the affinity of His‐tagged CcmL for QD was increased by 100 times compared to the wild‐type CcmL, which was responsible for the significantly improved assembly efficiency and homogeneity of PNCs [50].

The high affinity between histidine and metal ions can also be adopted to coupling molecules, such as multivalent chelators and tri‐nitrilotriacetic acid (tri‐NTA). For instance, a surface‐exposed His‐tag was fused to the C‐terminal end of the norovirus (NoV) VLPs. Then the imidazole rings were captured by the Ni‐loaded tri‐NTA. To carefully analyze the effect of the number of histidines, partially His‐tagged and fully His‐tagged NoV VLPs were respectively created. Both NTA‐bound functional molecules and hydrodynamic diameters of NoV VLPs are proportional to the number of decorated histidines [55]. The coupling of functional molecules on the PNC surface is therefore determined by the number of histidine residues, which can be explored for multi‐purposes.

Another example of imidazole‐mediated modification is the monofunctionalization of PNCs. Monofunctionalization is useful for assessing the contribution of an individual functional motif to PNCs. To achieve this, Li *et al*. inserted four histidine residues after His139 of SV40 VP1 to form a His‐tag exposed on the surface of SV40 VLPs. Through co‐assembly at a predicted optimal feeding ratio of His‐tagged to wild‐type SV40 VP1 (1:11) and then purification *via* affinity chromatography, the monofunctionalized VLP was generated. Each resultant VLP has 12 VP1 pentamers, with one functionalized and the other 11 non‐functionalized. The monofunctionalized VLPs served as a scaffold for precisely constructing photonic nanostructures of gold nanoparticles and QDs ( Fig.4) [51]. The study offers a strategy to break the natural symmetry of PNCs.

#### Modification mediated by tyrosine

Tyrosine is also a residue that can be utilized to cross‐link with other molecules. Finn's group generated inter‐subunit cross‐linking between tyrosine residues Y52 and Y103 using a complex Ni/tripeptide GGH/magnesium monoperoxyphthalate (MMPP). The complex was considered to withdraw electrons from tyrosine, and tyrosyl radical was created so that adjacent Y52 and Y103 were bridged. By quantitative analysis, when the complex Ni/GGH/MMPP was mixed at a ratio of 1:1:1 and added to CPMV VLPs at a stoichiometry of 100 equivalent of nickel per asymmetric (equals to two subunits), the best yield of cross‐linking was, therefore, obtained [56]. This method can strengthen the interactions between subunits, thereby enhancing the intensity and stability of VLPs.

Tyrosine‐mediated functionalization can also modify PNCs with metal ion complexes to construct magnetic resonance contrast agents with a high relaxation rate. Hooker *et al*., using MS2 VLPs as biomolecular scaffolds, firstly displayed aldehyde functional groups on the inner surface tyrosines (Tyr85) and outer surface amines (Lys106, Lys113, and N‐terminus). Through oxime condensation, about 90 heteropodal bis (hydroxypyridonate) terephthalamide ligands were linked to these sites. By attaching Gd‐chelates to the inner surface through tyrosine residues, the VLPs were endowed with better stability, water solubility, and a higher relaxation rate [57]. Similarly, at the same sites of MS2 VLPs (Lys106, Lys113, N‐terminus, Tyr85), Kovacs *et al*. modified the outer surface (up to 360 amino groups) by using PEG‐NHS esters through an orthogonal modification strategy, and Tyr 85 at inner surface undergoes rapid diazo coupling with p‐nitroaniline derivatives, generating VLPs encapsulating 50‒70 fluorochromes [58]. The dual‐surface‐modified VLPs can carry drugs while appending ligands to increase blood half‐life or targeting ability.

#### Modification mediated by unnatural amino acid

Unnatural amino acids often contain special groups that are available for a specific modification. In a study by Tong *et al*., up to 60 DNA strands were attached to each MS2 VLP *via* a periodate‐mediated reaction. Specifically, in order to load functional cargoes, they mutated Asn87 to Cys, that is, adding 180 sulfhydryl groups on the inner surface. For outer surface modification, firstly, *p*‐aminophenylalanine ( *pa*F) was introduced at position 19 using an amber stop codon suppression system, and then an aptamer was modified with N, N‐diethyl‐N′‐acylphenylene diamine moiety. NaIO_4_‐mediated oxidative coupling reaction was adopted to link the aptamer onto the outer surface ( Fig.4) [27]. In this way, functional MS2 VLPs were created by simultaneously decorating different targeting modules on the surface and encapsulating cargoes such as anticancer drugs or contrast agents in the inner cavity. The versatile VLPs can be employed as a potential carrier for targeted bioimaging and drug delivery.

Unnatural amino acids have also been introduced to PNCs for realizing multivalent functionalization. Patel *et al*. genetically replaced methionine residues of MS2 VLPs (mutant T16M‐M89L‐M109L) with methionine analogs for introducing unnatural amino acids azidohomoalanine (AHA) and homopropargylglycine (HPG) with the azide and alkyne sidechains, respectively, by a cell‐free protein synthesis method, while mutations at 89 and 109 were created to prevent functionalization at these two sites. Following azide‐alkyne click chemistry, an antibody fragment IM9scFv, a granulocyte‐macrophage colony‐stimulating factor (GM‐CSF), and CpG DNA all together were directly conjugated to the MS2 surface. As was determined by densitometry, the numbers of loaded functional motifs per 180‐mer MS2 VLP were *ca.* 50, 6 and 20 for IM9scFv, GM‐CSF, and CpG DNA, respectively [44]. Copper‐catalyzed azide‐alkyne cycloaddition (CuAAC) can also be used for cross‐linking in virtue of unnatural amino acids. In a study by Strable *et al*., azidohomoalanine and homopropargylglycine were replaced with methionine by reassignment of sense codons from HBV or Qβ capsid proteins. The azide group can participate in copper‐mediated addition to terminal alkynes. The result showed that HBV VLPs were sensitive to the conjugation of 4‐helix‐bundle spikes, while Qβ VLPs tolerated the introduction of triazole moieties. Therefore, Qβ VLPs were more suitable for CuAAC reaction [59]. This modification method, which firstly introduced azide or alkyne to PNCs by genetic modification and then establishes connection by CuAAC, is suitable for unnatural amino acids based on triazole linkage, enhancing the versatility of PNC modification, and simplifying the modification steps.

#### Biomineralization

Biomineralization is a process that ions in solution are converted into solid minerals in specific parts of organisms under certain physical or chemical conditions [60]. With a hollow interior, PNCs provide a confined environment for biomineralization. Ferritin nanoparticle is one of the representatives that are applied in mineralization. The ferritin cage has a nucleation site in the interior cavity with a high charge density. Douglas *et al*. took horse spleen ferritin (HSFn) as a reactor for mineralization induced by specific oxidative hydrolysis of Co(II). By electrostatic interactions between ferritin interior surface and Co(II), the dispersions of Co(O)OH were site‐specifically encapsulated inside ferritin cages, forming a mineral core constituted by over 2,000 Co atoms [61].

In addition to the inner cavity, the outer surface of PNCs can also be functionalized by biomineralization. For example, Du *et al*. investigated biomineralization on PNCs for improving the thermostability of FMDV VLPs. A calcium‐chelating peptide, W6, which can stimulate biomineralization to produce a CaP mineral shell on the PNCs, was introduced into the β‐loop of the VP1 protein of FMDV, resulting in 60 copies of W6 uniformly distributed on the surface of PNCs after self‐assembly. The biomineralization rate of VLPs‐W6 was greatly improved to 80% from 60% of the native VLPs. The CaP mineral shells not only improved the thermostability of FMDV VLPs and enhanced cellular uptake but also led to higher levels of specific neutralizing antibodies in guinea pigs after vaccination [62].

#### Self‐assembly

Self‐assembly is a non‐covalent approach for functionalizing PNCs quantitatively. Through self‐assembly, the quantity and variety of encapsulated or surface‐exposed functional modules can be precisely regulated. A typical paradigm is QD encapsulation in VLPs. Due to the restriction by the dimension of the inner cavity of VLPs, only one QD was encapsulated in SV40 VLPs [63,64]. On the other hand, the number of encapsulated cargoes can also be controlled by altering the electrostatic interactions between cargoes and capsid subunits. By mixing supercharged GFP (+36) molecules with engineered AaLS pentamers, the best encapsulation yield was precisely determined when the molar ratio reached 3 (AaLS monomer *vs.* GFP molecules), which was approximately 100 GFP (+36) molecules per capsid [65]. Moreover, electrostatic interactions between negatively charged single‐strand (ss) DNA and positively charged luminal surface of CCMV VLPs [66] have been investigated to encapsulate various enzymes‐DNA conjugates. The encapsulation of glucose oxidase (GOx)‐ssDNA complex, glucokinase (GCK)‐complementary sequence (cs) DNA complex, and GOx‐GCK dual‐enzyme complex not only stabilized the assembly of CCMV VLPs but also were developed as cascading enzyme systems. The molar ratio of the co‐encapsulated GOx‐GCK enzyme was calculated as 1:1.4 per capsid, indicating one combined GOx‐ssDNA and one or two GCK‐csDNA were encapsulated in each VLP [67]. Self‐assembly provides a flexible way to design multifunctional PNCs of higher‐order assemblies by utilizing the interactions between individual subunits and functional modules, where the functionalization can be quantitatively tuned.

In short, chemical modification generally refers to the attachment of single/multiple exogenous modules to functional groups on the inner/outer surface of PNCs under certain conditions. Among these modification methods, some have high specificity and high reaction efficiency, such as chelation of metal ions with certain groups, click chemistry (section of “modification mediated by unnatural amino acid”), *etc*. These “point‐to‐point” modification methods can accurately quantify modification based on the control of the number of modification sites. In contrast, some methods are relatively less specific, such as the NHS method and some tyrosine‐mediated modification, mineralization and modification methods based on the unique self‐assembling properties of PNCs. As such accurate quantitative control can hardly be realized, but the modification can be indirectly controlled by adjusting the reaction stoichiometry or order.

### Combination of genetic and chemical modification

In the case that individual modification methods cannot meet the need for designed functionalization, combining genetic and chemical methods will be necessary. Genetic engineering is often carried out to introduce single/multiple sites for chemical modification or to alter the features in the region of interest in VLPs to regulate the molecular recognition and the assembly of VLPs with functional modules.

**Figure 5:**
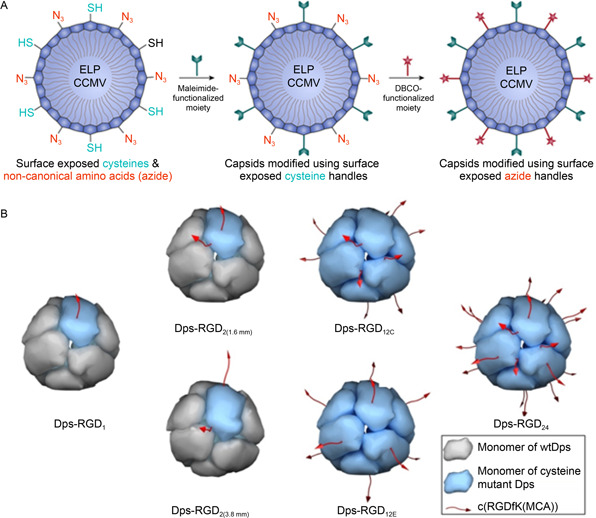
**Examples of combined genetic and chemical functionalization.** (A) Presentation of dual site‐selective handles on CCMV VLPs [68]. Copyright 2021 American Chemical Society. (B) Labeling of Dps PNCs with RGD peptides of different surface distribution patterns [69]. Copyright 2019 Wiley‐VCH.

In a recent study aiming to obtain double‐modified VLPs, an ELP was firstly fused to the N terminus of CCMV VLPs to construct the mutant, ELP‐CCMV, which not only improved the VLP stability but also caused conformation changes to expose the side chains of Cys59 residues on the VLPs. Next, azido phenylalanine (AzF) was introduced at the position of amino acid 65 (mutant K65AzF) using the amber suppression method, corresponding to one azide functional group per capsid protein. So, the outer surface of ELP‐CCMV has two kinds of available sites for modification, surface‐exposed cysteines and azido phenylalanines. Then the azide‐handles of VLPs were conjugated with sulfo‐Cyanine5‐DBCO *via* a strain‐promoted azide‐alkyne cycloaddition (SPAAC) reaction, while the VLPs were also modified with cysteine‐cell penetrating peptides (TAT) ( Fig.5). The resultant VLPs were taken up by HeLa cells with a significantly increased efficiency [68]. Interestingly, by combining genetic engineering, self‐assembly, and bioconjugation, precise functionalization of PNCs can be achieved. In one of our studies with the Dps PNCs, we covalently linked a well‐defined number of Arg‐Gly‐Asp (RGD) ligands onto the PNCs. Cysteines were introduced at carefully selected sites on the Dps surface by point mutation. Coassembly of the mutant wild‐type Dps subunits followed by affinity chromatography generated Dps PNCs bearing a specific number of surface cysteines. The RGD peptide was covalently linked to the PNCs by virtue of the sulfhydryl group of cysteine using an ultrahigh‐efficiency conjugation procedure. As a result, Dps PNCs displaying 1, 2, 12, and 24 RGD ligands with varied distribution patterns were created ( Fig.5). These models enabled the investigation of the effects of targeting ligand number and distribution on the tumor‐targeting performance of nanoparticles [69].

Genetic engineering, in guidance with structural information of PNCs, can regulate the binding of PNCs with other molecules and facilitate subsequent chemical functionalization. For instance, the charge properties of PNCs impose a crucial influence on biomineralization, so the residues in the inner cavity of PNCs can be genetically substituted to attract precursor ions to favor the site‐specific deposition of minerals. The CCMV VLPs have been employed as a template for the synthesis of iron oxide. By replacing six arginines and three lysines at the highly basic N‐terminal end with glutamic acids, the inner surface of CCMV VLPs was changed up to 3,240 units of charge. When the mutant VLPs were treated with iron ions and oxidized in the air, about 6,000 iron atoms were mineralized inside the cage [70]. In this study, the precisely designed electrostatic interactions on the inner surface played a critical role in the formation of iron oxide, which opened the possibility of templated mineralization in PNCs to fabricate hybrid bio‐nano materials, which is tunable in a genetic way.

In addition to the complementary working manner, combined genetic and chemical modification procedures are often carried out to endow PNCs with multiple independent functions. Ferritin is a representative PNC platform for constructing multifunctional nanoparticles. Li *et al*. reported a ferritin‐based probe that was able to image tumor cells in both fluorescence and nuclear magnetic resonance modes. To this end, a linker sequence (MGRGDSPSSSGGSGSGS) and an RGD peptide were fused to the N terminus of GFP‐ferritin fusion protein to construct the fluorescent PNCs with the tumor‐targeting RGD peptides. Then the mutant ferritin was loaded with iron oxide *via* mineralization by virtue of its intrinsic ion‐binding residues, with up to a theoretical value of 5,000 iron atoms per cage. In this way, the ferritin PNCs were functionalized with a well‐defined number of tumor‐targeting ligands, GFP, and ferrimagnetic nanoparticles. The multifunctional PNCs as fluorescent probes and magnetic resonance imaging contrast agents, showed effectiveness in recognizing α_v_β_3_‐integrin‐upregulated tumor cells with dual‐mode readouts [71].

In brief, combinatorial modification integrates the advantages of genetic and chemical modification methods greatly enriches the procedures for PNC functionalization. Generally, PNCs are first genetically modified, and then chemical decoration is executed on the genetically introduced handles. Therefore, combinatorial modification offers good flexibility and benefits the precise quantitative control of PNC functionalization.

## CONCLUSIONS

PNCs are a group of self‐assembling biomacromolecules that have been early recognized as natural nanomaterials. They have been modified by various strategies to fulfill various innovation attempts as well as practical applications. They have also been playing a unique role in bridging nanotechnology and biology [1,10,72,73]. Interestingly, the findings from nanobiological studies using PNCs are beginning to reward biology itself. For example, redesigned PNCs may work as artificial organelles in living cells in the field of synthetic biology. Designed functionalization is the key to realizing the potential of PNCs. In fact, modification of PNCs is not a new topic. And there are many reviews discussing this. However, few endeavors have been devoted to discussing this topic from a quantitative point of view. In this contribution, we have tried to do so with the emphasis on the precise quantitative control of PNC functionalization.

Having genetically encoded and highly symmetrical structures that can be solved at the atomic level, PNCs are one of the most suitable materials systems for precise modification compared with chemically synthesized macromolecules. Achievements of site‐specific decorated PNCs have made a leap forwards in a multi‐disciplinary context. This review summarizes the modification methods currently applied to PNCs and highlights the significance of quantitative functionalization. These methods for precise quantitative modification are categorized into three groups: genetic modification (point mutation and fragment fusion), chemical modification (covalent functionalization with cysteine, lysine, aspartic acid/glutamate, tyrosine, and unnatural amino acids, metal complexation with imidazole group from histidine, biomineralization and self‐assembly), and combined genetic and chemical modification. Combinations of different methods offer the flexibility to meet specific functionalization purposes.

Studies on precise quantitative modification are sometimes restricted by the complexities in the structures of proteins and functional molecules and the lack of quantitative measurement methods or testing instruments. These limitations exist not only for PNCs but also for most materials that require quantitative functionalization. Innovations of bioanalytical technologies in synthetic biology and artificial intelligence would promote breakthroughs in the precise quantitative functionalization of PNCs, thereby inspiring the applications of smart PNCs in many fields such as bioimaging, biosensing, vaccines, drug delivery, catalysis and so forth.

## COMPLIANCE WITH ETHICS GUIDELINES

The authors Quan Cheng, Xuan Wang, Xian‐En Zhang, Chengchen Xu and Feng Li declare that they have no conflict of interests.

This article is a review article and does not contain any studies with human or animal subjects performed by any of the authors.
